# Adaptive Evolution of RH5 in Ape *Plasmodium* species of the *Laverania* Subgenus

**DOI:** 10.1128/mBio.02237-17

**Published:** 2018-01-23

**Authors:** Lindsey J. Plenderleith, Weimin Liu, Oscar A. MacLean, Yingying Li, Dorothy E. Loy, Sesh A. Sundararaman, Frederic Bibollet-Ruche, Gerald H. Learn, Beatrice H. Hahn, Paul M. Sharp

**Affiliations:** a Institute of Evolutionary Biology, University of Edinburgh, Edinburgh, United Kingdom; b Centre for Immunity, Infection and Evolution, University of Edinburgh, Edinburgh, United Kingdom; c Department of Medicine, University of Pennsylvania, Philadelphia, Pennsylvania, USA; d Department of Microbiology, University of Pennsylvania, Philadelphia, Pennsylvania, USA; Columbia University

**Keywords:** *Laverania*, *Plasmodium falciparum*, RH5, basigin, chimpanzee, gorilla

## Abstract

*Plasmodium falciparum*, the major cause of malaria morbidity and mortality in humans, has been shown to have emerged after cross-species transmission of one of six host-specific parasites (subgenus *Laverania*) infecting wild chimpanzees (*Pan troglodytes*) and western gorillas (*Gorilla gorilla*). Binding of the parasite-encoded ligand RH5 to the host protein basigin is essential for erythrocyte invasion and has been implicated in host specificity. A recent study claimed to have found two amino acid changes in RH5 that “drove the host shift leading to the emergence of *P. falciparum* as a human pathogen.” However, the ape *Laverania* data available at that time, which included only a single distantly related chimpanzee parasite sequence, were inadequate to justify any such conclusion. Here, we have investigated *Laverania Rh5* gene evolution using sequences from all six ape parasite species. Searching for gene-wide episodic selection across the entire *Laverania* phylogeny, we found eight codons to be under positive selection, including three that correspond to contact residues known to form hydrogen bonds between *P. falciparum* RH5 and human basigin. One of these sites (residue 197) has changed subsequent to the transmission from apes to humans that gave rise to *P. falciparum*, suggesting a possible role in the adaptation of the gorilla parasite to the human host. We also found evidence that the patterns of nucleotide polymorphisms in *P. falciparum* are not typical of *Laverania* species and likely reflect the recent demographic history of the human parasite.

## IMPORTANCE

A number of closely related, host-specific malaria parasites infecting wild chimpanzees and gorillas have recently been described. The most important cause of human malaria, *Plasmodium falciparum*, is now known to have resulted from a cross-species transmission of one of the gorilla parasites. Overcoming species-specific interactions between a parasite ligand, RH5, and its receptor on host cells, basigin, was likely an important step in the origin of the human parasite. We have investigated the evolution of the *Rh5* gene and found evidence of adaptive changes during the diversification of the ape parasite species at sites that are known to form bonds with human basigin. One of these changes occurred at the origin of *P. falciparum*, implicating it as an important adaptation to the human host.

## INTRODUCTION

*Plasmodium falciparum* is the cause of the great majority of clinical cases of and deaths due to malaria in humans. This parasite has long been known to be only very distantly related to the other *Plasmodium* species that infect humans, leading to its classification in a separate taxon, *Laverania* (1), now recognized as a subgenus. For many years, the only other species described within the *Laverania* was *Plasmodium reichenowi*, a name applied nearly a century ago to parasites seen in the blood of wild-caught chimpanzees and gorillas that were morphologically indistinguishable from *P. falciparum* (2). The only strain of *P. reichenowi* that has been maintained and characterized was obtained from a captive chimpanzee (2). Over recent years, DNA sequences have been obtained from additional *Laverania* parasites (3–9), leading to the realization that what had previously been termed “*P. reichenowi*” in fact comprises six cryptic species (8, 10). Intriguingly, in the wild, these *Plasmodium* species appear to be strictly host specific: three (the newly named *P. gaboni* and *P. billcollinsi* and the original *P. reichenowi*) have only ever been found in chimpanzees, while the three others (*P. praefalciparum*, *P. adleri*, and *P. blacklocki*) have only been found in gorillas (8, 10). *P. praefalciparum* was so named because phylogenetic analyses revealed that it was the precursor of *P. falciparum* in humans (8). The relative dearth of genetic diversity in *P. falciparum* compared with that of chimpanzee *Laverania* species (11) and the apparent absence of other ape *Laverania* parasites infecting humans living in close proximity to African apes (12, 13) suggest that the human parasite arose from a single gorilla-to-human transmission in the recent past. These observations raise questions of what determines host specificity and why ape-to-human transmissions are not more common.

In this context, parasite proteins that mediate specific and essential interactions with host proteins are of obvious interest. One such interaction is that between reticulocyte-binding protein homologue 5 (RH5), a *P. falciparum* ligand, and its receptor on the erythrocyte surface, human basigin (BSG). Binding of RH5 to BSG was shown to be essential for invasion of erythrocytes by all strains of *P. falciparum* tested (14). Subsequently, it was shown that *P. falciparum* RH5 bound chimpanzee BSG with lower affinity than human BSG and failed to bind gorilla BSG (15). *P. falciparum* strains (of human origin) have been found to infect captive chimpanzees and bonobos (*Pan paniscus*), but not gorillas (5, 7, 16, 17). Thus, the host tropism of *P. falciparum* seemed to correlate with the strength of RH5-BSG interactions, raising the possibility that adaptation at the *Rh5* locus might have been an important step in the origin of the human parasite (15).

Comparing gene sequences between *P. falciparum* and *P. reichenowi*, Otto et al. found no indication of adaptive evolution (i.e., nonsynonymous differences fixed by natural selection) in *Rh5* (18). In contrast, a more focused analysis using a different approach has reported evidence of adaptive amino acid changes in both RH5 and BSG, leading to the conclusion that “Positive selection underlies the species-specific binding of *Plasmodium falciparum* RH5 to basigin” (19). However, close scrutiny of the data used and the results obtained seriously undermines this conclusion. First, the particular amino acid changes highlighted in basigin were irrelevant to the evolution of *P. falciparum* (detailed later). Second, the data set of *Rh5* sequences analyzed was inadequate for an attempt to identify positive selection during the emergence of *P. falciparum*. Third, it has since been shown that the *Rh5* sequences that were analyzed are not related in the manner that might have been anticipated (11). Due to horizontal gene transfer into the gorilla parasite that gave rise to *P. falciparum*, the *P. reichenowi* and *P. falciparum Rh5* gene sequences are more than seven times more divergent than the average across other genes compared between these two species. As a consequence, it is necessary to compare *P. falciparum* with ape *Laverania* species other than *P. reichenowi* to elucidate whether positive selection has indeed played a role in the recent evolutionary history of the *Rh5* gene and the binding of RH5 to human basigin. Here, we describe *Rh5* sequences from six ape *Laverania* species and show that multiple residues in RH5 have been subject to episodic selection during the divergence of the ape *Laverania*. However, only a single change, not previously identified, could possibly be related to the origin of *P. falciparum*.

## RESULTS

### Reexamination of previous analyses of *Rh5* and basigin.

In the context of a genome-wide analysis, Otto et al. (18) compared the ratios of nonsynonymous to synonymous nucleotide changes between polymorphisms (*pN*/*pS*) and interspecies differences (evolutionary changes [*dN*/*dS*]), using five *Rh5* gene sequences from *P. falciparum* and one from the *P. reichenowi* strain CDC. This McDonald-Kreitman (M-K) test (20) assumes that synonymous changes are neutral and can provide evidence of adaptive evolution if there is a relative excess of nonsynonymous differences between species. However, for *Rh5*, there was an excess (albeit nonsignificant) of nonsynonymous polymorphisms within *P. falciparum*. This could reflect the demographic history of *P. falciparum*, which is likely to have undergone massive population expansion as the numbers of humans increased over recent millennia. Population expansion can lead to an accumulation of slightly deleterious mutations, which makes it more difficult to detect any nonsynonymous differences between species that have been fixed by adaptation.

**FIG 1:**
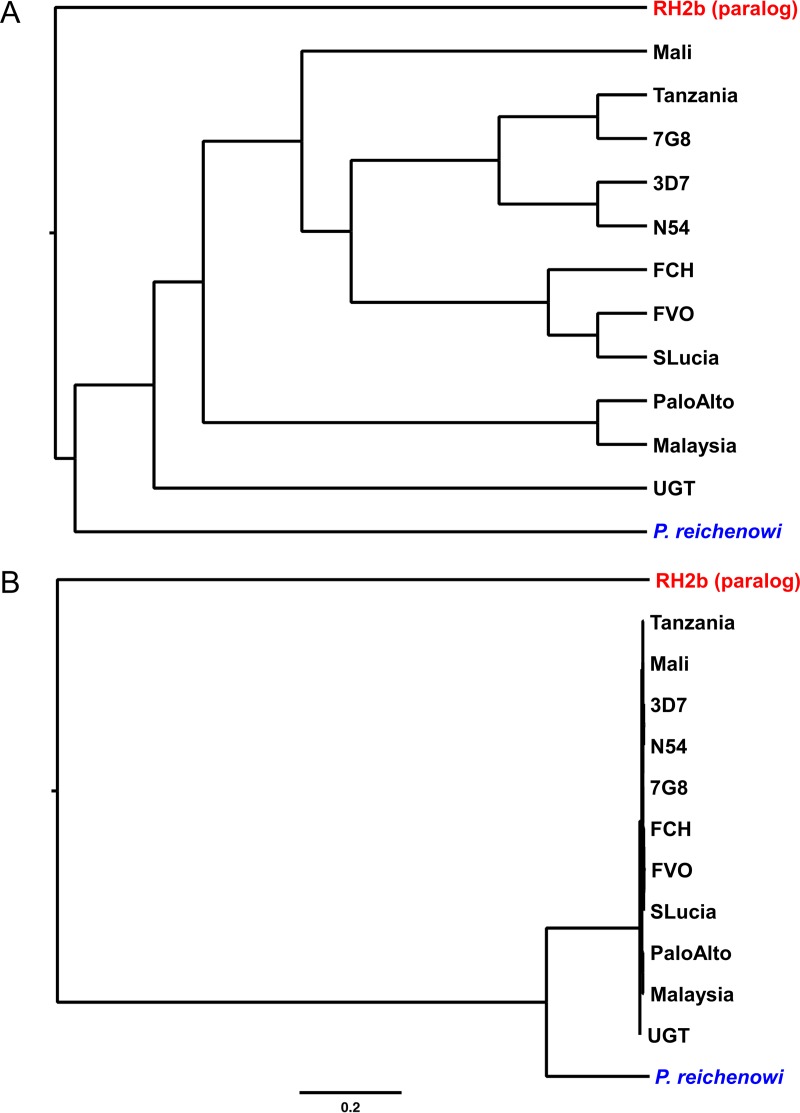
Relationships among RH5 sequences analyzed by Forni et al. (19). (A) Phylogeny as depicted in reference 19. (B) Maximum-likelihood analysis of the same protein sequences, with branch lengths drawn to scale; the bar indicates 0.2 amino acid replacements per site. In each panel, the top sequence (red) is *P. falciparum* RH2b, a paralogue of RH5, and the bottom sequence (blue) is *P. reichenowi* strain CDC RH5; the other sequences (black) are RH5 from various *P. falciparum* strains.

**FIG 2:**
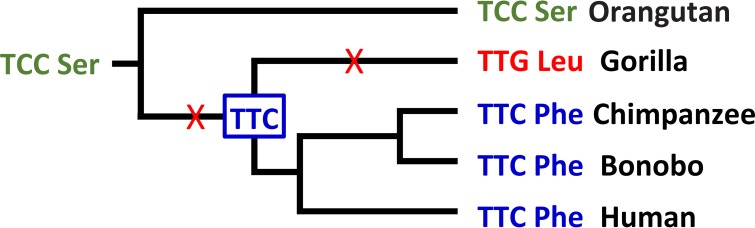
Evolution of codon 27 in the basigin (BSG) genes of hominids. The amino acids seen in these species require a minimum of two nonsynonymous substitutions; the most parsimonious locations of these changes in the phylogeny are shown. Colors indicate different codons. The TCC codon at the root of this tree is inferred by comparison with the BSG genes of Old World monkeys.

Forni et al. (19) examined the ratio of nonsynonymous to synonymous changes among 12 *Rh5* sequences, 11 from *P. falciparum* and one from the same *P. reichenowi* strain, CDC, used by Otto et al. (18); in the absence of an *Rh5* sequence from an outgroup species, they added the sequence of a paralogue, *Rh2b* from *P. falciparum*, to root the tree of *Rh5* sequences. However, this set of sequences does not have any power to detect positive selection on RH5 during the origin of *P. falciparum*. First, the RH2b sequence used as an outgroup has only 27% amino acid sequence identity with the RH5 sequences, and in the alignment used by Forni et al., approximately one-third of all codons in *Rh5* are aligned against gaps in the *Rh2b* sequence (19). Second, the *P. falciparum* sequences differed on average by only 0.2% of nucleotides, whereas the *P. reichenowi* sequence differs from that of *P. falciparum* by 16.3%. However, as we show in Fig. 1, the relationships of the sequences were depicted by Forni et al. (Fig. 2A in reference 19) in a phylogenetic tree with distorted branch lengths.

Despite this apparent lack of useful information, the analyses performed by Forni et al. detected two codons (190 and 447) as putatively subject to positive selection on the branch leading to *P. falciparum* (19). Codon 190 is TAT (encoding Tyr) in the 11 *P. falciparum* sequences, compared to ATA (Ile) in *P. reichenowi* CDC, while codon 447 is TGG (Trp) in all strains of *P. falciparum* and AAT (Asn) in *P. reichenowi* CDC. Thus, at both codons, there have been (at least) three substitutions during the divergence of the two species; at codon 447 all three would have been nonsynonymous, while at codon 190, at least two of the three were nonsynonymous. As noted above, it is to be expected that the *Rh2b* sequence is too divergent to be used as an effective outgroup. In fact, in the alignment used by Forni et al. (19), both of these codons were opposite a gap inserted in the *Rh2b* sequence, confirming that it was not possible to estimate from these data what the sequence of the common ancestor of *P. falciparum* and *P. reichenowi* might have been. Thus, there was no information available to determine which (if any) of these nucleotide changes might have occurred on the branch leading from that ancestor to *P. falciparum*, as opposed to the branch to *P. reichenowi*.

To investigate the evolution of basigin (BSG), Forni et al. compared sequences from 28 primate species and identified two codons (codons 27 and 102) where amino acid changes were putatively brought about by positive selection (19). However, again, close scrutiny of the data indicates that neither of these changes can be linked to the emergence of *P. falciparum* in humans. First, the 28 species of primates included 19 species of Old World and New World monkeys, 3 species of prosimians, and 2 species of Asian apes, none of which has been found to be naturally infected by *Laverania* species, and thus, most of the data were not relevant to the recent evolution of the parasites infecting African apes. In fact, codon 102 encodes His in gorillas, chimpanzees, and humans, and any changes at this BSG site prior to the last common ancestor of the African apes can have had no bearing on the recent interactions of *Laverania* species with this receptor. While the amino acid encoded by codon 27 does vary among the African apes, it is also difficult to see how this can be linked to any interactions with *Laverania* RH5. BSG site 27 encodes Phe in both humans and chimpanzees but Leu in gorillas, and thus, a parasite adapted to infecting chimpanzees would seem to be better preadapted to infecting humans than a gorilla parasite. The likely points when, during hominin divergence, the nonsynonymous substitutions at this site occurred (Fig. 2) are not obviously correlated with changes in RH5. Furthermore, if BSG had adapted because of its interaction with RH5, it might be expected that this would reflect selection to disable the interaction, in order to inhibit *Plasmodium* infection; clearly such selection has not been effective in preventing *P. falciparum* from infecting humans.

### Evolution of RH5 in *Laverania* species.

**FIG 3:**
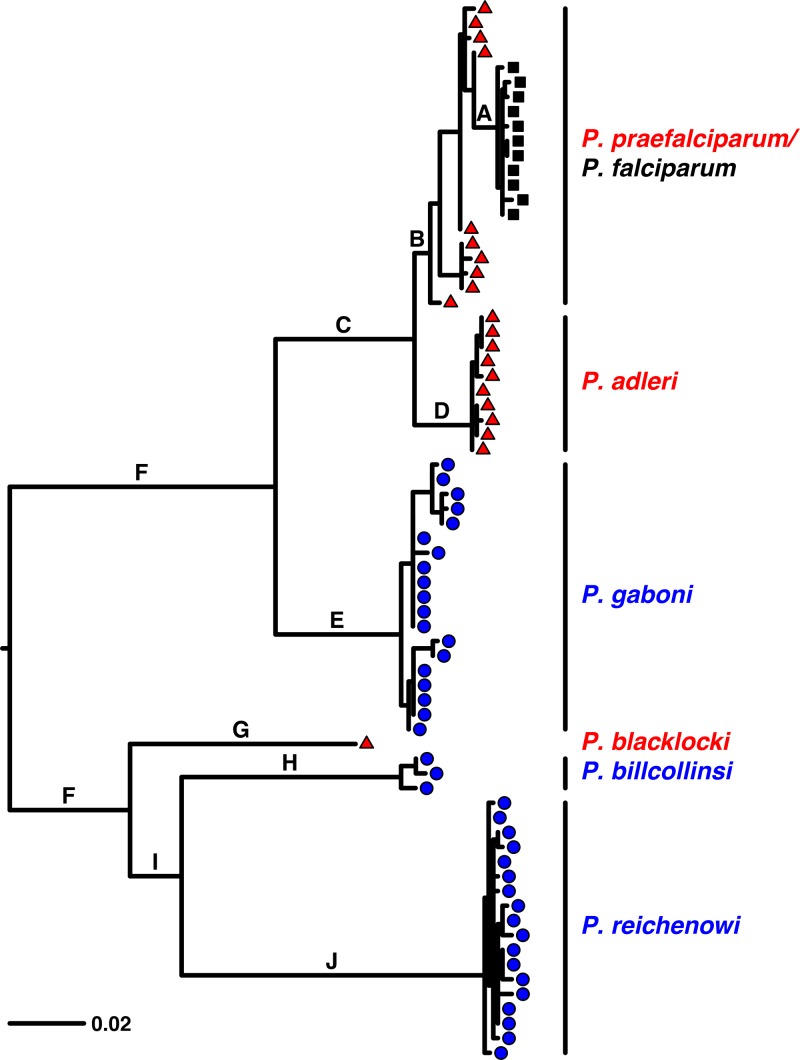
Evolutionary relationships among *Rh5* genes from *Laverania* species. Species infecting gorillas are shown in red (each sequence is represented by a triangle), species infecting chimpanzees in blue (circles), and *P. falciparum* isolates from humans in black (squares). Internal branches between species are labeled to allow them to be identified in the text. The scale bar indicates 0.02 nucleotide substitutions per site.

We have determined an additional 27 partial *Rh5* gene sequences from ape fecal and blood samples and combined these with our previous data. As shown recently (11), the phylogenetic relationships among *Laverania Rh5* sequences differ from those derived from mitochondrial DNA (8) or other nuclear gene sequences (10). Compared to the phylogenies of other genes, in the *Rh5* phylogeny, the *P. praefalciparum* clade of gorilla parasites that encompasses *P. falciparum* has moved from being closely related to *P. reichenowi* (from chimpanzees) to being closely related to *P. adleri*, another gorilla parasite (Fig. 3); this is most simply interpreted as resulting from horizontal gene transfer from an ancestor of *P. adleri* into an ancestor of *P. praefalciparum* (11).

We obtained multiple alleles from all ape *Laverania* species except *P. blacklocki*, for which we amplified only one short sequence. For comparative purposes, we added the 11 *P. falciparum* genome sequences analyzed previously (19). For *Rh5*, *P. falciparum* had the lowest nucleotide diversity among the *Laverania* species (Table 1), although the disparity was not as large as seen in genome-wide comparisons with two of the chimpanzee parasites, *P. reichenowi* and *P. gaboni* (11). Notably, in the short alignment (846 nucleotides; see Materials and Methods), *P. praefalciparum* strains exhibited more than four times more diversity than *P. falciparum*, consistent with the latter having emerged from a bottleneck at transmission from gorillas to humans. The pattern of diversity in *P. falciparum* is also unusual in that all of the polymorphisms are nonsynonymous (Table 1); a test of heterogeneity of the relative numbers of nonsynonymous and synonymous polymorphisms among species is significant (*P* < 0.05) for both the long (1,272 nucleotides; see Materials and Methods) and short alignments but becomes nonsignificant if *P. falciparum* is excluded. This suggests that the patterns of polymorphisms in the ape parasite sequences are a better null model to use in M-K tests of the divergence between species.

**TABLE 1:** Polymorphisms in the *Rh5* gene in *Laverania* species

Species (host)^a^	Value in indicated alignment^b^							
	Long				Short			
	No. of sequences	π	No. of:		No. of sequences	π	No. of:	
			*pN*	*pS*			*pN*	*pS*
*P. falciparum* (H)	11	0.0017	9	0	11	0.0020	8	0
*P. praefalciparum* (G)	2	0.0008	1	0	10	0.0092	16	5
*P. adleri* (G)	4	0.0014	1	2	10	0.0024	2	3
*P. gaboni* (C)	10	0.0059	10	8	19	0.0073	10	6
*P. blacklocki* (G)	0				1			
*P. billcollinsi* (C)	2	0.0066	2	6	3	0.0068	2	6
*P. reichenowi* (C)	6	0.0044	7	6	18	0.0039	10	5

a Natural hosts are humans (H), chimpanzees (C), or gorillas (G).

b Long and short alignments are defined in Materials and Methods. π, nucleotide diversity per site; *pN*, nonsynonymous polymorphisms; *pS*, synonymous polymorphisms.

In comparisons among pairs of closely related ape parasite species, direction of selection (DoS) values are always positive, indicating a relative excess of nonsynonymous substitutions among species. However, the only pairwise comparisons yielding statistically significant values in M-K tests were those involving *P. billcollinsi* (Table 2); this results from the unusually large fraction of synonymous changes among polymorphisms in that species (Table 1). An M-K test across the entire phylogeny of ape parasites (excluding *P. falciparum*) using the long alignment is formally significant (see Table S2 in the supplemental material) but susceptible to error because the number of synonymous substitutions may have been underestimated, particularly on the long branch (branch F in Fig. 3) spanning the root of the tree. For the short alignment, where more sequences are available, the test result is highly significant (Table 2), to an extent that seems less likely to be explained by unscored synonymous substitutions. Thus, these data suggest an excess of nonsynonymous changes among the fixed differences among species, consistent with adaptive evolution of *Rh5*.

**TABLE 2:** Comparisons of nonsynonymous and synonymous polymorphisms and divergence^a^

Comparison	No. of^b^:				DoS value^c^	M-K test^d^
	*pN*	*pS*	*dN*	*dS*		
All ape parasite species	40	25	198	59	0.155	<0.001
*P. falciparum* vs *P. praefalciparum*	24	5	2	1	−0.161	0.48
*P. falciparum* vs *P. adleri*	10	3	17	3	0.081	0.66
*P. falciparum* vs *P. gaboni*	18	6	42	15	−0.013	1.0
*P. praefalciparum* vs *P. adleri*	18	8	12	2	0.165	0.45
*P. praefalciparum* vs *P. gaboni*	26	11	37	13	0.037	0.81
*P. adleri* vs *P. gaboni*	12	9	45	12	0.218	0.08
*P. reichenowi* vs *P. billcollinsi*	12	11	70	19	0.265	0.017
*P. reichenowi* vs *P. blacklocki*	10	5	68	28	0.042	0.77
*P. billcollinsi* vs *P. blacklocki*	2	6	64	20	0.512	0.006

a Values were obtained from the short alignment.

b *pN*, nonsynonymous polymorphisms; *pS*, synonymous polymorphisms; *dN*, nonsynonymous substitutions; *dS*, synonymous substitutions.

c Direction of selection (DoS) values were calculated as *dN*/(*dN* + *dS*) − *pN*/(*pN* + *pS*) (34).

d *P* values from the M-K test (23) are shown, as determined using a Fisher exact test.

We applied the recently developed branch-site unrestricted statistical test for episodic diversification (BUSTED) (21) to our long *Rh5* alignment for six species, excluding *P. blacklocki* because only a shorter sequence was available for that species. Designating all branches as foreground, i.e., allowing for positively selected sites on any branch in one model compared to no positively selected sites in the alternative model, provided strong evidence of episodic selection (*P* = 0.0011). With this approach, it is difficult to formally test the significance of selection at individual codons, but evidence ratios (ERs) derived as two times the site-specific log likelihood ratio between the alternative models are believed to provide a measure of support for each site (21). In this analysis, eight codons had ERs greater than 4 (each potentially significant using a *P* value of <0.05), but none had ERs greater than 6. Thus, while the overall evidence for episodic selection seems strong, the evidence for each of these candidate codons was relatively weak. When the *P. blacklocki* sequence was added to the analysis, the overall evidence of episodic selection remained strong (*P* = 0.0042), while the support for each candidate codon weakened further, with ERs for two (381 and 442) dropping below 4.

**FIG 4:**
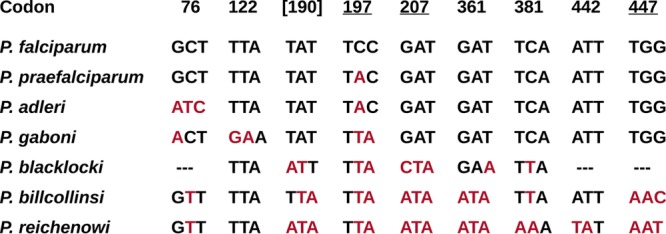
Codons in *Rh5* identified as candidate sites of episodic selection during the diversification of the *Laverania*. Eight codons (excluding codon 190, in square brackets) were identified in the current analysis, including three (underlined codons 197, 207, and 447) that are contact residues involved in the binding of *P. falciparum* to human BSG (see Fig. 5). One of these (codon 197) changed during the recent divergence of *P. falciparum* from *P. praefalciparum*. Nucleotides differing from the *P. falciparum* sequence are shown in red. Note that the *P. blacklocki* sequence only covers codons 106 to 381.

**FIG 5:**
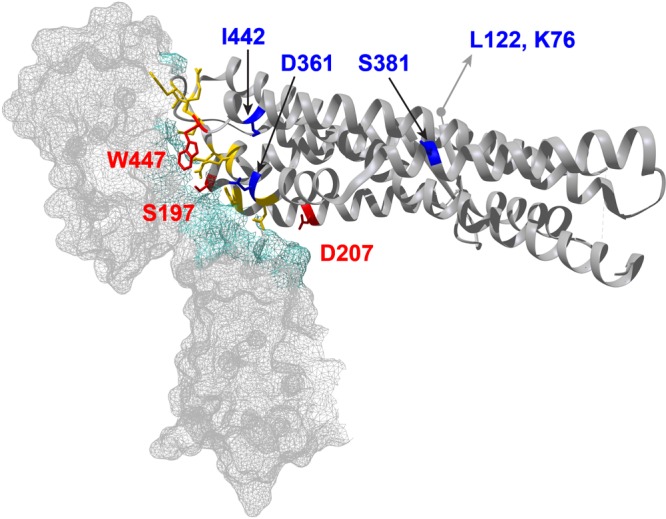
Cocrystal structure of *P. falciparum* RH5 (ribbons) in complex with human BSG (mesh), modified from reference 22. Eight amino acids in RH5 encoded by sites identified here as candidates contributing to the signal of episodic selection across the *Laverania* phylogeny are highlighted either in red (if they are known binding sites between *P. falciparum* and human BSG) or in blue; two (K76 and L122) are within the N-terminal region, whose structure is unknown.

The eight candidate codons and their character states in the different species are shown in Fig. 4. Two codons were previously claimed to be under putative positive selection (19). One (codon 190) was not identified here, and neither it nor the other (codon 447) could have played a role in the emergence of *P. falciparum*, because both are conserved among the human parasite and its three closest relatives (Fig. 4). The eight codons identified here have undergone at least 26 nucleotide substitutions across the *Laverania Rh5* phylogeny, and by tracing the most likely character states at each ancestral node within the phylogeny, it is possible to assign 18 substitutions to particular branches (Table S3). The largest number of substitutions (at least 6 and perhaps as many as 10) appears to have occurred on branch F (Fig. 3); this is perhaps unsurprising, because it is the longest branch, spanning the root of the phylogeny. One substitution, a nonsynonymous change at the second position of codon 197, is inferred to have occurred on branch A (Fig. 3), which separates *P. falciparum* from its precursor, *P. praefalciparum*. Mapping the eight encoded residues onto the structure of *P. falciparum* RH5 in complex with human BSG (22) reveals that three are at sites within RH5 that are known to bind BSG (Fig. 5). These include the serine at site 197.

## DISCUSSION

The malaria parasite *P. falciparum* became widespread among humans following a cross-species jump of a gorilla parasite, perhaps within the last few thousand years (23); understanding the host-parasite interactions that allowed that event is of obvious interest. A recent analysis of the evolution of the RH5-BSG ligand-receptor pair concluded that the data “support the hypothesis that positive selection at these genes drove the host shift leading to the emergence of *P. falciparum* as a human pathogen” (19). However, consideration of the *Rh5* gene sequence data set used previously reveals that it was inadequate and should not have been used to support such claims: Forni et al. analyzed *Rh5* sequences from only two species (*P. falciparum* and *P. reichenowi*) and, thus, *a priori* had no power to infer on which branch from their common ancestor any changes had occurred (19). Another problem for the previous analysis was that, unbeknown to its authors, the *Rh5* gene was subject to horizontal gene transfer during recent *Laverania* evolution, such that the *P. reichenowi* sequence is unusually distantly related to the gorilla parasite that gave rise to *P. falciparum* (11). As a consequence, any differences observed between *P. reichenowi* and *P. falciparum* are much less likely to have occurred in the recent ancestry of the human parasite.

We have obtained a much more comprehensive data set, including *Rh5* gene sequences from all six known species of ape *Laverania* parasites related to *P. falciparum*. We find that the two residues in RH5 identified by Forni et al. (19) as subject to positive selection are in fact conserved among *P. falciparum* and the three other species that are most closely related for this genomic region (Fig. 4); thus, these differences between *P. falciparum* and *P. reichenowi* cannot have been at all relevant to the host shift leading to the emergence of *P. falciparum*. However, our data set points to positive selection at other sites in RH5 during the diversification of the *Laverania*, including one residue (site 197) implicated in binding to host BSG (22). Interestingly, this residue appears to have changed subsequent to the cross-species transmission from gorillas that gave rise to *P. falciparum* (see below). We note that methods looking for evidence of adaptive evolution from unusually high rates of nonsynonymous substitution in particular codons are only likely to identify sites that have undergone recurrent changes across the sequence sets analyzed. Such methods can be very useful when applied to fast-evolving RNA viruses like HIV-1 (21) but are likely to overlook many adaptive changes that occur only once. For example, apart from site 197, in our sample, there are five fixed amino acid differences between the *P. falciparum* and *P. praefalciparum* RH5 sequences, any of which could have been an adaptation involved in the origin of *P. falciparum*, but these were not flagged as under positive selection, presumably because there are few other nonsynonymous substitutions at these codons across the *Laverania* phylogeny.

Other methods, such as the M-K test, compare patterns of nucleotide polymorphisms and divergence to look for evidence of adaptive changes across the gene as a whole (20). When this method has been applied to *P. falciparum* in the past, the comparison has necessarily been between polymorphisms within *P. falciparum* and divergence from a single sequence from the only other available species, *P. reichenowi*. These tests have often found an excess of nonsynonymous differences within species, which has then been interpreted as evidence of selection to maintain amino acid polymorphisms in *P. falciparum* (see, for example, references 18 and 24). However, very low levels of synonymous nucleotide polymorphisms were found in some of the earliest studies of *P. falciparum* genetic variation (25). At least for the *Rh5* gene, sequences from *Laverania* species infecting apes now show that the pattern of polymorphisms in *P. falciparum* is unusual, with the ratio of synonymous to nonsynonymous differences being much higher in each of the ape parasites (Table 1). This difference between *P. falciparum* and the other *Laverania* species might reflect different selection pressures on the human parasite but is perhaps most simply explained by the recent origin of *P. falciparum* via a host jump from gorillas (8). Following a bottleneck at cross-species transmission (11), the *P. falciparum* population expanded, and it presumably continued to do so over historical time as its host population also grew. During population expansion, slightly deleterious nonsynonymous changes that would otherwise be eliminated by natural selection can accumulate (a recent population bottleneck was invoked previously to explain the lack of synonymous polymorphisms in *P. falciparum*, but without knowledge of the cause of the bottleneck [25]). In this light, it seems more appropriate to use polymorphism data from the ape parasites as the comparator in M-K tests: for the two chimpanzee parasites for which genome-wide polymorphism data are available, *P. reichenowi* and *P. gaboni*, the nucleotide diversity levels are much higher than in *P. falciparum* (11), suggesting that their demographic history has been more stable. When we used the ape parasite sequences in M-K tests of *Rh5* sequences, there were indications of an excess of nonsynonymous changes between species.

In conclusion, two different approaches suggest that amino acid changes in RH5 have been subject to selection during the divergence of *Laverania* parasites infecting apes in Africa. Given the essential role of RH5 in mediating erythrocyte invasion, it is possible that changes at these sites influence the strict chimpanzee or gorilla host specificity seen among these parasites in the wild. One of the sites in RH5 that was identified as having been under positive selection (site 197) is of particular interest because it is a BSG contact residue (Fig. 5) that has undergone an amino acid change during the recent divergence of *P. falciparum* from its gorilla-infecting precursor (Fig. 4). However, in a survey of global *P. falciparum* diversity (the Pf3k project; https://www.malariagen.net/projects/pf3k), this codon is polymorphic. While all of 1,490 samples from Africa have the Ser codon, 457 of 920 (50%) samples from Southeast Asia have a Tyr codon, similar to the gorilla parasites *P. praefalciparum* and *P. adleri*. Noting that *P. falciparum* clearly originated in Africa, the Tyr-to-Ser change could have been an adaptation necessary for the initial colonization of the new (human) host but, nevertheless, of suboptimal fitness. The reversion to Tyr, which has spread in Southeast Asia, may have been enabled by a compensatory change elsewhere in RH5 or in one of the other parasite proteins (CyRPA, Ripr, and P113) that are now known to form a complex with RH5 that enables BSG interaction (26–29). We also note that residue 100 in human BSG, which binds to the *P. falciparum* RH5 site 197, is conserved as Gln among humans and chimpanzees, and while this site is polymorphic among western gorillas, the most common allele encodes Gln (data from reference 30). Thus, the change in *P. falciparum* RH5 does not simply reflect adaptation to a fixed difference at this site between gorilla and human BSG. Clearly, further studies, including structural analyses of ape *Laverania* RH5 proteins and their respective host BSGs, are needed to elucidate the possible role of RH5 site 197 in the origin of malignant malaria in humans.

## MATERIALS AND METHODS

### *Rh5* gene sequences.

The *Rh5* gene of *P. falciparum* comprises 527 codons, with a 221-nucleotide intron interrupting codon 22 (31). Full-length *Rh5* gene sequences are available from two *P. reichenowi* and two *P. gaboni* genome sequences (11, 18). We have previously determined partial *Rh5* gene sequences from *Laverania* species obtained from fecal samples of wild-living chimpanzees and gorillas (11), 31 of which were used here. Using the same limiting dilution PCR approach (see reference 11 for details), we focused on species underrepresented in the earlier data set and obtained an additional 27 sequences; 13 of these were instances where the length of previously characterized alleles could be extended, while 14 represent new alleles amplified from fecal (*n* = 2) and blood (*n* = 4) samples using additional *Rh5* primer sets (details available upon request; see Table S1 in the supplemental material for a list of the sequence data). We added the 11 sequences of human *P. falciparum* used by Forni et al. (19). Two different data sets were analyzed: a long alignment for 35 sequences (1,272 nucleotides, covering codons 115 to 521), and a short alignment for 72 sequences (846 nucleotides, covering codons 115 to 379). The sequences were aligned using Muscle (32), with manual correction.

10.1128/mBio.02237-17.1 TABLE S1  Description of *Rh5* gene sequences analyzed. Download TABLE S1, PDF file, 0.1 MB. Copyright © 2018 Plenderleith et al. 2018 Plenderleith et al. This content is distributed under the terms of the Creative Commons Attribution 4.0 International license.

10.1128/mBio.02237-17.2 TABLE S2  Nonsynonymous and synonymous polymorphisms and divergence across the long alignment. Download TABLE S2, PDF file, 0.1 MB. Copyright © 2018 Plenderleith et al. 2018 Plenderleith et al. This content is distributed under the terms of the Creative Commons Attribution 4.0 International license.

10.1128/mBio.02237-17.3 TABLE S3  Assignment of substitutions in *Rh5* to branches of the *Laverania* phylogeny. Download TABLE S3, PDF file, 0.1 MB. Copyright © 2018 Plenderleith et al. 2018 Plenderleith et al. This content is distributed under the terms of the Creative Commons Attribution 4.0 International license.

The numbers of nonsynonymous and synonymous substitutions evident as polymorphisms within species (*pN* and *pS*) or substitutions between species (*dN* and *dS*) were counted with DnaSP (33) and compared with the M-K test (20), using a Fisher exact test, and the direction of selection (DoS) statistic was calculated as *dN*/(*dN* + *dS*) − *pN*/(*pN* + *pS*) (34). Under the assumption that synonymous mutations are neutral, a significant result in the M-K test may reflect an excess of nonsynonymous differences fixed between species, where DoS is positive, or an excess of nonsynonymous polymorphisms, where DoS is negative. These values were computed for a comparison involving all parasite species and for comparisons of pairs of closely related species, i.e., those lying on the same side of the root of the phylogenetic tree.

### Phylogenetic analyses.

Phylogenetic trees were generated by maximum-likelihood methods implemented in PhyML (35). Nucleotide sequences were analyzed using the general time-reversible model with gamma-distributed rate variation among sites (GTR+G); protein sequences were analyzed using the Jones-Taylor-Thornton model with gamma-distributed rate variation and a class of invariant sites (JTT+G+I).

For the initial tests of selection, one sequence was chosen from each species: exon 2 sequences were taken from published genomes where available and otherwise chosen at random from the longest sequences available for that species (Table S1). To test for episodic selection on the *Rh5* gene across the *Laverania* phylogeny, we used BUSTED as implemented in the HyPhy package (21). This method allows the ratio of nonsynonymous/synonymous substitutions (dN/dS) to vary among branches and among sites and tests for evidence of selection occurring on a specified set of so-called “foreground” branches (which can include all branches). It compares the likelihood of (i) a model of selection that allows a *dN*/*dS* ratio of >1 at a fraction of sites on all branches with that of (ii) a null model that does not allow a *dN*/*dS* ratio of >1 on the foreground branches; twice the difference in log likelihoods is compared with a chi-square distribution with 2 degrees of freedom. This method also estimates evidence ratios, measuring the support for positive selection at each codon on the foreground branches.

Sites within the cocrystal structure of *P. falciparum* RH5 in complex with human basigin were visualized using the UCSF Chimera package, version 1.10.2 (36).

### Accession number(s).

The nucleotide sequences of *Laverania Rh5* sequences are available under GenBank accession numbers KT824390 to KT824423 and MF356538 to MF356555.
