# Supplementary material for: Adaptive Evolution of RH5 in Ape Plasmodium species of the Laverania Subgenus
Source: mBio. 2018 Jan 23;9(1):e02237-17. doi: 10.1128/mBio.02237-17 (PMC5784257; doi:10.1128/mBio.02237-17)
Supplement: TABLE S1 [file mbo001183680st1.pdf]

TABLE S1 Description of *Rh5* gene sequences analyzed

Sample name	Species	Accession/ID[a]	Source	Length (bp)	Alignment[b]
BQggg60_RH5_SGA6.11	*P. adleri*	KT824390	published SGA sequence	827	n/a
BQggg61_RH5_SGA6.11	*P. adleri*	(KT824390)	published SGA sequence	827	n/a
EKggg1182_FB_SGA8.8	*P. adleri*	KT824396	updated SGA sequence	1253	long
NDggg3120_RH5_SGA6.11	*P. adleri*	(KT824390)	published SGA sequence	827	n/a
SAggg3066_FA_SGA30.G10	*P. adleri*	MF356539	new SGA sequence	1349	n/a
SAggg3066_FA_SGA30.G8	*P. adleri*	MF356538	new SGA sequence	1349	n/a
SAggg3066_FB_SGA30.F4	*P. adleri*	MF356542	new SGA sequence	1253	long
SAggg3066_FL_SGA100.3	*P. adleri*	MF356541	new SGA sequence	1773	long
SAggg3066_FL_SGA30.F3	*P. adleri*	MF356540	new SGA sequence	1814	long, 1ps
SAggg3157_RH5_SGA100.1	*P. adleri*	MF356543	new SGA sequence	827	n/a
GTptt503_RH5_SGA5.6	*P. billcollinsi*	KT824404	published SGA sequence	833	n/a
SYptt20_FB_SGA5.1	*P. billcollinsi*	KT824413	updated SGA sequence	1371	n/a
SYptt20_FL_SGA	*P. billcollinsi*	MF356544	updated SGA sequence with new accession	1726	long, 1ps
GTggg119_RH5_SGA10.3	*P. blacklocki*	KT824399	published SGA sequence	830	1ps
Pf_7G8	*P. falciparum*	EU433391	published SGA sequence	1581	long
Pf_FCH	*P. falciparum*	KI927815	RH5 exon 2 extracted from published contig	1517	long
Pf_FVO	*P. falciparum*	KI925024	RH5 exon 2 extracted from published contig	1517	long
Pf_Malaysia	*P. falciparum*	KI927479	RH5 exon 2 extracted from published contig	1517	long
Pf_Mali	*P. falciparum*	KI925502	RH5 exon 2 extracted from published contig	1517	long
Pf_NF54	*P. falciparum*	KE123747	RH5 exon 2 extracted from published contig	1517	long
Pf_PaloAlto	*P. falciparum*	KI927255	RH5 exon 2 extracted from published contig	1517	long
Pf_SLucia	*P. falciparum*	KE123478	RH5 exon 2 extracted from published contig	1517	long
Pf_Tanzania	*P. falciparum*	KI926311	RH5 exon 2 extracted from published contig	1517	long
Pf_UGT5.1	*P. falciparum*	KE124438	RH5 exon 2 extracted from published contig	1517	long
Pf3D7	*P. falciparum*	PF3D7_0424100	published genome	1581	long, 1ps
GTptt314_RH5_SGA5.4	*P. gaboni*	KT824403	published SGA sequence	827	n/a
GTptt604_RH5_SGA5.1	*P. gaboni*	KT824405	published SGA sequence	827	n/a
GTptt604_RH5_SGA5.3	*P. gaboni*	KT824406	published SGA sequence	827	n/a
KApts1680_RH5_SGA30.1	*P. gaboni*	KT824407	published SGA sequence	827	n/a
PgSY37	*P. gaboni*	PGSY37_0424100	published genome	1562	long
PgSY75	*P. gaboni*	PGSY75_0424100	published genome	1581	long, 1ps
SYpte37_RH5_SGA5.1	*P. gaboni*	KT824408	published SGA sequence	827	n/a
SYpte57_RH5_SGA60.12	*P. gaboni*	(KT824408)	published SGA sequence	827	n/a
SYpte66_FB_SGA5.1	*P. gaboni*	MF356545	updated SGA sequence with new accession	1242	long
SYptt13_FB_SGA5.1	*P. gaboni*	KT824410	updated SGA sequence	1252	long
SYptt5_FB_SGA5.1	*P. gaboni*	KT824415	updated SGA sequence	1226	long
SYptt61_FB_SGA5.1	*P. gaboni*	MF356546	new SGA sequence	1252	long
SYptt61_FB_SGA5.4	*P. gaboni*	KT824416	updated SGA sequence	1252	long
SYptt61_FB_SGA5.7	*P. gaboni*	MF356547	new SGA sequence	1239	long
SYptt63_FB_SGA5.4	*P. gaboni*	KT824417	updated SGA sequence	1238	long
SYptt63_RH5_SGA60.10	*P. gaboni*	KT824418	published SGA sequence	827	n/a
SYptt65_RH5_SGA6.9	*P. gaboni*	KT824420	published SGA sequence	827	n/a
SYptt65_RH5_SGA60.1	*P. gaboni*	(KT824408)	published SGA sequence	827	n/a
TCptt18_FB_SGA5.8	*P. gaboni*	KT824423	updated SGA sequence	1252	long
DSggg24_RH5_SGA6.7	*P. praefalciparum*	KT824395	published SGA sequence	869	n/a
GTggg10_RH5_SGA6.4	*P. praefalciparum*	KT824397	published SGA sequence	848	n/a
GTggg118_RH5_SGA10.4	*P. praefalciparum*	KT824398	published SGA sequence	844	n/a
GTggg119_FB_SGA5.4	*P. praefalciparum*	MF356549	new SGA sequence	1290	long

GTggg119_FL_SGA	*P. praefalciparum*	MF356548	updated SGA sequence with new accession	1924	long, 1ps
GTggg119_RH5_SGA5.8	*P. praefalciparum*	KT824400	published SGA sequence	869	n/a
SAggg3157_FA_SGA30.A10	*P. praefalciparum*	MF356550	new SGA sequence	1327	n/a
SAggg3157_RH5_SGA30.B7	*P. praefalciparum*	MF356552	new SGA sequence	848	n/a
SAggg3157_RH5_SGA30.D6	*P. praefalciparum*	MF356553	new SGA sequence	848	n/a
SAggg3157_RH5_SGA30.D7	*P. praefalciparum*	MF356551	new SGA sequence	848	n/a
DGptt540_FB_SGA5.1	*P. reichenowi*	MF356554	updated SGA sequence with new accession	1259	long
DGptt540_FB_SGA5.5	*P. reichenowi*	KT824394	updated SGA sequence	1259	long
DGptt540_RH5_SGA30.1	*P. reichenowi*	KT824392	published SGA sequence	833	n/a
DGptt540_RH5_SGA30.10	*P. reichenowi*	KT824391	published SGA sequence	833	n/a
DGptt540_RH5_SGA30.3	*P. reichenowi*	KT824393	published SGA sequence	833	n/a
DGptt540_RH5_SGA30.4	*P. reichenowi*	(KT824419)	published SGA sequence	833	n/a
GTptt212_RH5_SGA5.1	*P. reichenowi*	KT824401	published SGA sequence	833	n/a
GTptt212_RH5_SGA5.4	*P. reichenowi*	KT824402	published SGA sequence	833	n/a
PrCDC	*P. reichenowi*	PRCDC_0421300	published genome	1608	long, 1ps
PrSY57	*P. reichenowi*	PRSY57_0421300	published genome	1608	n/a
SYptt15_RH5_SGA60.5	*P. reichenowi*	KT824411	published SGA sequence	833	n/a
SYptt47_RH5_SGA10.5	*P. reichenowi*	KT824414	published SGA sequence	833	n/a
SYptt64_RH5_SGA60.5	*P. reichenowi*	KT824419	published SGA sequence	833	n/a
SYptt7_FB_SGA5.1	*P. reichenowi*	MF356555	new SGA sequence	1250	long
SYptt7_RH5_SGA60.12	*P. reichenowi*	KT824421	published SGA sequence	833	n/a
TCptt18_FB_SGA20.6	*P. reichenowi*	KT824422	updated SGA sequence	1398	long
TCptt18_RH5_SGA60.11	*P. reichenowi*	KT824424	published SGA sequence	833	n/a
TCptt18_RH5_SGA60.2	*P. reichenowi*	KT824425	published SGA sequence	833	n/a

[a]An accession number in brackets indicates that the sample had a sequence identical to the sequence associated with the accession number, but was from a different sample than the one referred to in the database entry. It is considered an independent sequence but does not have a separate accession number.
[b]'long' indicates inclusion in the 'long alignment', '1ps' indicates inclusion in the 'one sequence per species' alignment. All sequences were included in the short alignment and the alignment for the tree in Fig. 3. Further details of the different alignments are given in the Methods.
