# Supplementary material for: Adaptive Evolution of RH5 in Ape Plasmodium species of the Laverania Subgenus
Source: mBio. 2018 Jan 23;9(1):e02237-17. doi: 10.1128/mBio.02237-17 (PMC5784257; doi:10.1128/mBio.02237-17)
Supplement: TABLE S2 [file mbo001183680st2.pdf]

Table S2 Nonsynonymous and synonymous polymorphism and divergence across the long alignment

Comparison	P_N	P_S	D_N	D_S	DoS[a]	M-K[b]
All ape parasite species	21	22	248	67	0.299	0.017
P. falciparum vs	10	0	12	4	-0.250	0.14
P. falciparum vs *P. adleri*	10	2	22	4	0.013	1.0
P. falciparum vs *P. gaboni*	19	8	51	20	0.015	1.0
P. praefalciparum vs *P. adleri*	2	2	20	6	0.269	0.28
P. praefalciparum vs *P. gaboni*	11	8	51	19	0.150	0.26
P. adleri vs *P. gaboni*	11	10	56	18	0.233	0.06
P. reichenowi vs *P. billcollinsi*	9	12	101	26	0.367	<0.001

[a]DoS calculated as $D_N/(D_N+D_S) - P_N/(P_N+P_S)$ (34).
[b]P values from the M-K test (23) in a Fisher exact test.
