# Supplementary material for: Adaptive Evolution of RH5 in Ape Plasmodium species of the Laverania Subgenus
Source: mBio. 2018 Jan 23;9(1):e02237-17. doi: 10.1128/mBio.02237-17 (PMC5784257; doi:10.1128/mBio.02237-17)
Supplement: TABLE S3 [file mbo001183680st3.pdf]

TABLE S3 Assignment of substitutions in *Rh5* to branches of the *Laverania* phylogeny

Branch[a]	A	B	C	D	E	F	G	H	I	J	Sum[b]
Probable[c]	1	0	2	1	2	6[d]	0	0	2	4	18
Possible[e]	0	2	0	2	2	4[d]	1	1	1	1	8

[a]Letters refer to inter-species branches in the *Laverania* phylogeny in Fig. 3.
[b]Total number of inferred substitutions; only the eight codons at which adaptive evolution was inferred to have occurred are included (i.e., those in Fig. 4, with the exception of codon 190).
[c]Probable substitutions are those unambiguously assigned to one branch by maximum parsimony. For example, the substitutions in the first two positions of codon 122 (Fig. 4) are inferred to have occurred on branch E.
[d]Two of these substitutions occurred at sites where information from *P. blacklocki* is missing; the possibility that these substitutions occurred in branch I cannot be excluded.
[e]Possible substitutions are those that may have occurred on different pairs of branches. For example, the two substitutions at the first position of codon 207 (Fig. 4) may have occurred on branches F and G, on branches F and I, or on branches G and I.
